# Acoustofluidics Powered Synthesis of Bacterial Cellulose

**DOI:** 10.1021/acssuschemeng.5c07599

**Published:** 2025-11-18

**Authors:** Jikai Zhang, Katie Gilmour, Meng Zhang, Yunhong Jiang, Peter Arnold, Huiling Ong, Qiang Wu, Maryam Parsa, Ran Tao, Jingting Luo, Yongqing Fu

**Affiliations:** † School of Engineering, Physics and Mathematics, 5995 Northumbria University at Newcastle, Newcastle upon Tyne NE1 8ST, U.K.; ‡ Living Construction Group, School of Geography and Natural Sciences, 5995 Northumbria University at Newcastle, Newcastle upon Tyne NE1 8ST, U.K.; § Key Laboratory of Optoelectronic Devices and Systems of Ministry of Education and Guangdong Province, College of Physics and Optoelectronic Engineering, 47890 Shenzhen University, Shenzhen 518060, China

**Keywords:** Surface acoustic waves, Bacterial cellulose, Acoustofluidics, Biomanufacturing, Acoustic stimulation, Ambient synthesis

## Abstract

Bacterial cellulose (BC) possesses unique structural and functional properties including good water retention capacity, biocompatibility, and chemical stability and is currently widely used across various industries. However, its production typically relies on prolonged cultivation at around 30 °C, and it remains a major challenge to synthesize it at ambient temperature due to reduced bacterial activity and limited oxygen availability. In this study, we explored the application of acoustic wave technology to enhance BC production under static cultivation at ambient temperature, as an alternative to the conventional 30 °C incubation method. Acoustic wave induced acoustic radiation, streaming, and localized heating effects improved bacterial growth, nutrient distribution, and oxygen availability, thereby overcoming the limitations of low-temperature environments and barriers for access to oxygen at air–liquid interfaces. The acoustic wave agitated group exhibited enhanced bacterial proliferation, with BC pellicles achieving comparable (if not higher) yields to those of the 30 °C control group and significantly outperforming the ambient temperature control group. Structural analysis confirmed that acoustic stimulation preserved the nanoscale morphology and material integrity of BC, with mechanical properties similar to those of BC from the 30 °C control group. Furthermore, acoustic wave treatment reduced energy consumption approximately 10-fold and carbon emissions by over 90% compared to those of the routine 30 °C incubation process, demonstrating its outstanding energy efficiency and environmental sustainability. This work presents a novel approach for enabling BC biosynthesis at ambient temperature and provides new mechanistic insights into the acoustic regulation of microbial metabolism and material assembly.

## Graphical abstract

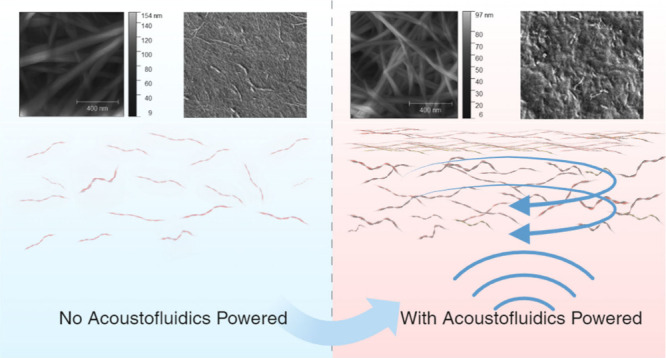

## 1 Introduction

Bacterial cellulose (BC) is a highly pure extracellular polysaccharide produced by bacterial strains such as *Komagataeibacter xylinus*, exhibiting the same chemical composition as that of plant cellulose but being superior in terms of nanofiber structure, mechanical strength, water retention, and biocompatibility.^1–4^ These features make BC attractive for wide-range applications in food, packaging, biomedicine, and tissue engineering.^5^ A widely used method for BC production is static fermentation, where thick pellicles are formed at the air–liquid interface under nonagitated conditions.^6^ However, this process is hindered by severe limitations in oxygen and nutrient diffusion through the pellicles, resulting in extended culture times and reduced productivity.^7^ The continuous thickening of BC further exacerbates this barrier effect, restricting access to the most active biosynthesis zone and ultimately slowing down the cellulose production.^8^

To enhance BC pellicle production, various solutions have been explored, such as controlling fermentation parameters,^9^ adopting different fermentation modes,^10^ and applying bioreactor technology.^11^ Among them, rotary^12^ and airlift^13^ bioreactors have been applied to improve nutrient and oxygen distributions. Rotating disk bioreactors periodically expose the BC pellicle production area to air and culture medium, ensuring a sufficient oxygen supply while effectively absorbing nutrients from the medium. However, during the rotation process, the shear force generated by the motion may affect the alignment of BC pellicle nanofibers, affecting its mechanical properties.^14,15^ Airlift bioreactors enhance microbial growth by lifting the liquid culture medium with gas flow, ensuring a uniform oxygen distribution. However, the airflow induced bubbles may alter the BC pellicle’s morphology, leading to formation of porous membrane structures.^16^ Additionally, fluid circulation often causes the BC pellicle to form fragmented or irregular structures.^17,18^ Currently there is an urgent need for active and effective methods for enhancing the BC synthesis.

Recent studies revealed that ultrasonics and acoustic waves (especially surface acoustic waves or SAWs) could be applied to modulate bacterial growth behavior, either enhancing or inactivating the bacterial growth.^19^ These results underscore the ability of the acoustic waves or SAWs to precisely control microenvironmental fluid dynamics and influence the biological reactions and metabolic processes, including nutrient uptake, metabolism, and spatial organization.^20–23^ In-depth analysis of recent studies^24,25^ revealed that SAWs modulated the fluid dynamics around bacterial cells, thereby enhancing mass transfer and oxygen availability, which are two critical factors in BC production. Previously ultrasonics have been utilized to modify BC functions.^26–28^ However, their direct applications and mechanisms in stimulating bacterial activity and enhancing BC biosynthesis have not yet been reported.

In this work, SAWs, for the first time, were employed to generate continuous acoustic wave stimulation of *K. xylinus*, aiming to accelerate bacterial growth and enhance BC production rates in the ambient environment and at room temperature. Figure 1 illustrates the SAW-based platform which was used in this study, where a chamber containing the bacterial culture was placed above the interdigital transducer (IDT) structure. In this setup, acoustic stimulation was applied to the bacterial culture through a SAW device placed beneath the growth chamber. Acoustic streaming generated by SAWs induces shear flow within the culture medium.^29–31^ It produces radiation forces and agitation effects on the particles or cells within the liquid, enhancing the distribution of nutrients and oxygen, while simultaneously stimulating bacterial growth and metabolic activity.^32–35^ This induced shear flow within the liquid addresses one of the primary limitations of static fermentation, i.e., the restricted oxygen supply. By maintaining the desirable characteristics of static cultivation (such as high crystallinity, unified cellulose network, and low-shear environment) while introducing acoustic wave stimulation, streaming, and localized heating effects, SAW technology can effectively and significantly shorten cultivation time, improve energy efficiency, and yield cellulose films with controllable and consistent mechanical properties. Accordingly, this study aimed to evaluate whether SAW-assisted static cultivation at ambient temperature can achieve BC yield and film quality similar to those obtained under the conventional 30 °C cultivation process, thereby demonstrating its potential as a sustainable and reliable approach for bacterial cellulose production.

**Figure 1:**
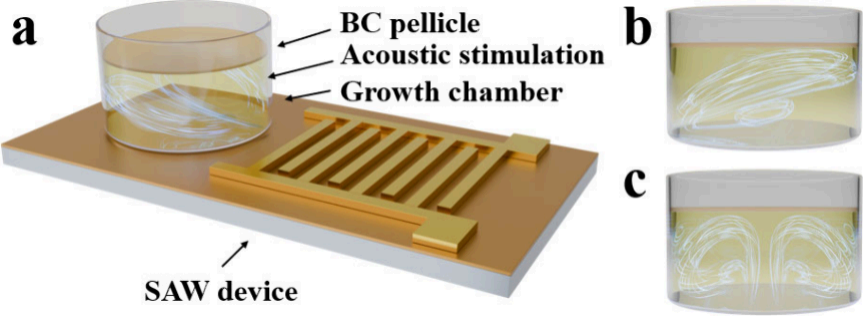
SAW-based acoustofluidic system and its working principle in BC pellicle formation. (a) Schematic of the SAW device setup, where a growth chamber containing the bacterial culture medium is placed on top of the IDT structure. (b) Front view and (c) side view of the acoustic streaming patterns generated within the culture medium under SAW excitation, illustrating enhanced fluid motion and improved nutrient and oxygen distribution that promote BC pellicle formation.

## 2 Methods

### 2.1 SAW Device and Experimental Setup

A ZnO/Al SAW device was used in this study, and its fabrication process was reported in our previous study.^36^ The SAW testing setup is shown in Figure S1 in the Supporting Information. The resonant frequency of the SAW device was ∼9.5 MHz, and the interdigital transducer (IDT) electrode had a wavelength of 300 μm. A signal generator (AFG1062, Tektronix) was used to generate the radio frequency (RF) signal of the resonant frequency. After being amplified by a power amplifier (75A250, Amplifier Research), the RF signals were input to the IDTs of the SAW device to generate SAWs. A power meter (9104, Racal) was used to measure the power input to the IDTs of the SAW device. The culture medium temperature was measured by using a thermocouple (2029T, Digitron). Temperature measurements were carried out in a 20 °C incubator (Medline Scientific AAH21172K), with the growth chamber placed on the surface of the SAW device. An ultrasonic gel (UGEL250, Anagel, Turkey) was used as a coupling agent. The medium temperature was recorded at different SAW input powers of 0.05, 0.1, 0.5, and 1 W.

### 2.2 BC Growth and Preparation

The cellulose producing bacteria, *K. xylinus* DSM 2325, was used in these experiments and obtained from culture collection DSMZ (Germany). Yeast Extract Peptone Dextrose (YPD) medium was prepared using the standard composition. A solution of 20 g/L peptone, 10 g/L yeast extract, and 20 g/L d-dextrose (herein for consistency, we use the term glucose in the following text) was prepared with autoclave sterilization separately. For the BC culture, the cells were initially grown in YPD medium containing 1% (v/v) cellulase from *Trichoderma reesei* (Sigma-Aldrich, C2730) at 30 °C and 200 rpm for 3 days to reach an optical density at 600 nm (OD_600nm_) between 0.9 and 1.2. The culture was then pelleted and washed in fresh YPD medium 4 times before being resuspended in the same volume of YPD to remove the cellulase. The pellicles were grown by adding the starter culture to fresh YPD medium at a ratio of 1:100 and incubating at 21 and 30 °C without applying SAWs (i.e., placed in temperature-controlled incubators set to be 21 and 30 °C, respectively, Medline Scientific AAH21172K) and also at 20 °C with SAW-treatment (cultivated in a 20 °C incubator, during which the SAW application at a power of 0.5 W increased the medium temperature to ∼21 °C). The total duration was 120 h. The key cultivation conditions for these three experimental groups are summarized in Table 1. To obtain BC pellicles of different sizes for analysis, fermentation was conducted in 25 and 100 mL beakers, with YPD culture medium volumes of 10 and 25 mL, respectively. The BC pellicles were harvested after 48, 72, 96, and 120 h of culture. They were washed with sterile deionized (DI) water to remove medium components and then submerged and washed with gentle agitation (60 rpm) with 500 mL of 70% ethanol for 1 h in a 500 mL beaker. Afterward, the BC pellicles were washed twice with equal volumes of sterile DI water for 30 min each. The samples were then collected for further drying or analysis. Three independent biological replicates (with separate cultures) were performed for each condition.

**Table 1:** Summary of Experimental Scenarios and Cultivation Conditions

Group	Temperature (°C)	SAW Stimulation	Culture Volume	Purpose
Ambient static control	21	No	Beaker, 10 or 25 mL	Baseline static culture at room temperature
30 °C static control	30	No	Beaker, 10 or 25 mL	Conventional incubation benchmark
SAW-treated group	20 (incubator base) → ∼21 (with SAW)	Yes (9.5 MHz, 0.5 W continuous)	Beaker, 10 or 25 mL	SAW stimulation applied under ambient conditions

### 2.3 Characterization of BC Products and Structures

#### 2.3.1 Growth Monitoring

A culture sample (0.1 mL) was taken daily from the beaker, for the full 120 h. Once the BC pellicles were formed from day two onward, they were harvested and lysed with 0.9 mL of phosphate-buffered saline (PBS) containing 1% (v/v) cellulase on a rocker at 4 °C. The culture medium and lysed BC were then serially diluted 10-fold with PBS. Then a 10 μL dilution of each (from 1 × 10^3^ to 1 × 10^9^) was inoculated onto YPD 1% (w/v) agar plates and incubated at 30 °C until colonies were formed. For each dilution, five technical replicate plates were prepared, and only the plates containing 30–300 colonies were counted to minimize the counting bias. The CFUs (colony-forming units) of *K. xylinus* were calculated daily for both the pellicles and supernatants, with each condition tested in triplicate.

#### 2.3.2 Glucose Utilization Assessment

Utilization of glucose was monitored using a 3,5-dinitrosalicylic acid (DNSA) assay, which measures the reduced sugar level (i.e., glucose). For sample preparation, 0.1 mL of supernatant from the growth medium was boiled in a 1.5 mL microcentrifuge tube at 95 °C for 15 min by a dry block heater (BH-250, Cole-Parmer), followed by centrifugation at 14,000 × *g* for 5 min, and the supernatant was used for the reducing sugar assay. The DNSA stock solution comprised 10 g/L 3,5-dinitrosalicylic acid, 300 g/L sodium potassium tartrate tetrahydrate, and 20% (v/v) 2 M NaOH. For this assay, 50 μL of sample, 450 μL of DI water, and 500 μL of DNSA stock solution were placed in a 1.5 mL microcentrifuge tube and boiled at 95 °C for 20 min. The absorbance of all of the samples was measured with a laser beam wavelength of 540 nm by the microplate reader (Spark, Tecan). YPD medium with varied glucose concentrations (i.e., 20, 10, 5, 1, 0.2, and 0.1 g/L) was used to determine the standard curves, and YPD medium containing 0 g/L of glucose served as a blank control.

#### 2.3.3 Wet and Dry Weight Testing

The weight of the saturated BC pellicle after washing was recorded as the wet weight. Each sample was measured five times to obtain the average wet weight. A standardized drying and blotting procedure was implemented to ensure consistency in the wet weight measurement. After harvesting, the BC pellicles were placed on a preweighed filter paper for 2 min to allow excess surface water to drain naturally. A second piece of filter paper was applied with consistent pressure to remove residual surface moisture. Then the BC samples were weighed immediately after blotting. The dry weight was measured after the sample was dried at 30 °C to a constant weight, usually after 3–4 days, confirmed by repeated weighing until the difference between two consecutive measurements was less than 0.1 mg. The sample was measured 5 times to obtain the average value.

#### 2.3.4 Morphology and Microstructure Analysis

A scanning electron microscope (SEM, MIRA3, TESCAN) was used to characterize surface morphology and microstructures of the BC. Before SEM characterizations, the dried BC pellicle was placed on a sample stage and coated with platinum using a sputter coater (Q150V ES Plus, Quorum) to avoid any charging effect.

An atomic force microscope (AFM, Dimension Edge, Bruker, Germany) was used to characterize the surface morphology of the BCs. AFM measurement was conducted in tapping mode. The Gwyddion software (version 2.63) was used to obtain the 3D AFM images. The diameters of fibers were obtained from AFM images of each fiber and with average readings of more than five fibers using the ImageJ software (Version 1.54f, USA). The average values and standard deviations are given in each figure.

X-ray diffraction (XRD, SmartLab NS, Rigaku, Japan) was applied to characterize the crystalline structures of the BC samples. XRD patterns of dried BC nanofibers were recorded using the Cu Kα radiation source (with a wavelength of λ = 1.54 Å, a voltage of 40 kV and a current of 30 mA). Samples were scanned in the 0–40° 2θ-range at scan speed of 0.5° min^–1^. The crystallinity (*X*_c_) was calculated based on X-ray diffraction measurements, using the equation $X_{c}\left(\%\right)=\frac{S_{c}}{S_{t}}\times 100$ , where *S*_c_ is the sum of the net area of crystalline peaks and *S*_t_ is the total area (including both crystalline and amorphous phases).

The pellicles were analyzed using the Fourier transform infrared spectroscopy (FTIR, Alpha, Bruker Corporation, USA) to characterize chemical structures and identify functional groups present in the bacterial cellulose. The transmittance FTIR signals were recorded with the visualization software OPUS (Bruker Corporation, USA).

#### 2.3.5 Mechanical Property Testing

Mechanical properties of BC samples were tested using an Instron tensile tester (68TM-50, Instron). Three strips (1 cm × 5 cm) were cut from each BC film according to the standard of ASTM D883. A total of nine samples were tested per treatment condition. The tensile strength of this material was measured at an extension rate of 20 mm/min. Young’s modulus (*E*, MPa) and elongation at break (ε, %) were automatically obtained from the Instron software (Bluehill 2, Instron, France). The photo of the mechanical testing process is shown in Figure S2 in the Supporting Information.

### 2.4 On-site Energy Consumption and Greenhouse Gas (GHG) Emission

The energy consumption (*E*) was calculated using the following equations:^37^

E=P×T 1

$$E_{\mathrm{per‐g}}=\frac{E}{M_{\mathrm{dry‐weight}}}$$ 2

where *E* is total energy consumption (in kWh), *P* is operating power (in W) of the incubator or SAW device, *T* is the culture time (in hours), *E*_per‑g_ is energy consumption per gram of BC (in kWh/g). The operating power of the incubator was measured by using an energy monitor (KTEM02BL, Ketotek, China), and that of the SAW device was measured by using a power meter (9104, Racal Dana).

The carbon emissions (*C*, in kg CO_2_e) was calculated using the following equation:^38^

C=E×I 3

$$C_{\mathrm{per‐g}}=\frac{C}{M_{\mathrm{dry‐weight}}}$$ 4

where *E* is the energy consumption (in kWh) and *I* is the carbon intensity (in kg CO_2_e/kWh). The global average carbon intensity is assumed as 0.4 kg CO_2_e/kWh, and *C*_per‑g_ is carbon emissions per gram of BC (in kg CO_2_e/g). In this study, GHG emissions refer to direct CO_2_-equivalent emissions associated with electricity consumption during cultivation experiments.

### 2.5 Statistical Analysis

The data obtained in this study are presented as mean values ± standard error of the mean and were analyzed using one-way analysis of variance (ANOVA).^39^ Pairwise comparisons between different groups were performed using the Tukey’s post hoc test.^40^ All of the cultures were conducted in triplicate, and all of the experiments were repeated at least three times. In the figures, statistical significance levels were defined as follows: *p* ≤ 0.05 (*), *p* ≤ 0.01 (**), and *p* ≤ 0.001 (***), while “ns” denotes a nonsignificant difference (*p* > 0.05).

## 3 Results and Discussion

### 3.1 Aerobic Bacterial Growth Behavior Modulation with SAWs

The impact of SAWs on the *K. xylinus* growth rate was quantified by colony-forming unit (CFU) counts in the culture medium (10 mL per sample; Figure 2a) and within the BC pellicle (Figure 2b). The SAW-treated group exhibited significantly higher bacterial proliferation in the medium compared with the 21 °C static control group, despite the identical incubation temperatures (Figure 2a). This control temperature was chosen because SAW application at the selected power level induced a ∼1 °C temperature rise in the medium, making 21 °C a more accurate thermal baseline for the SAW treatment (see the temperature data in Supplementary Figure S3).

**Figure 2:**
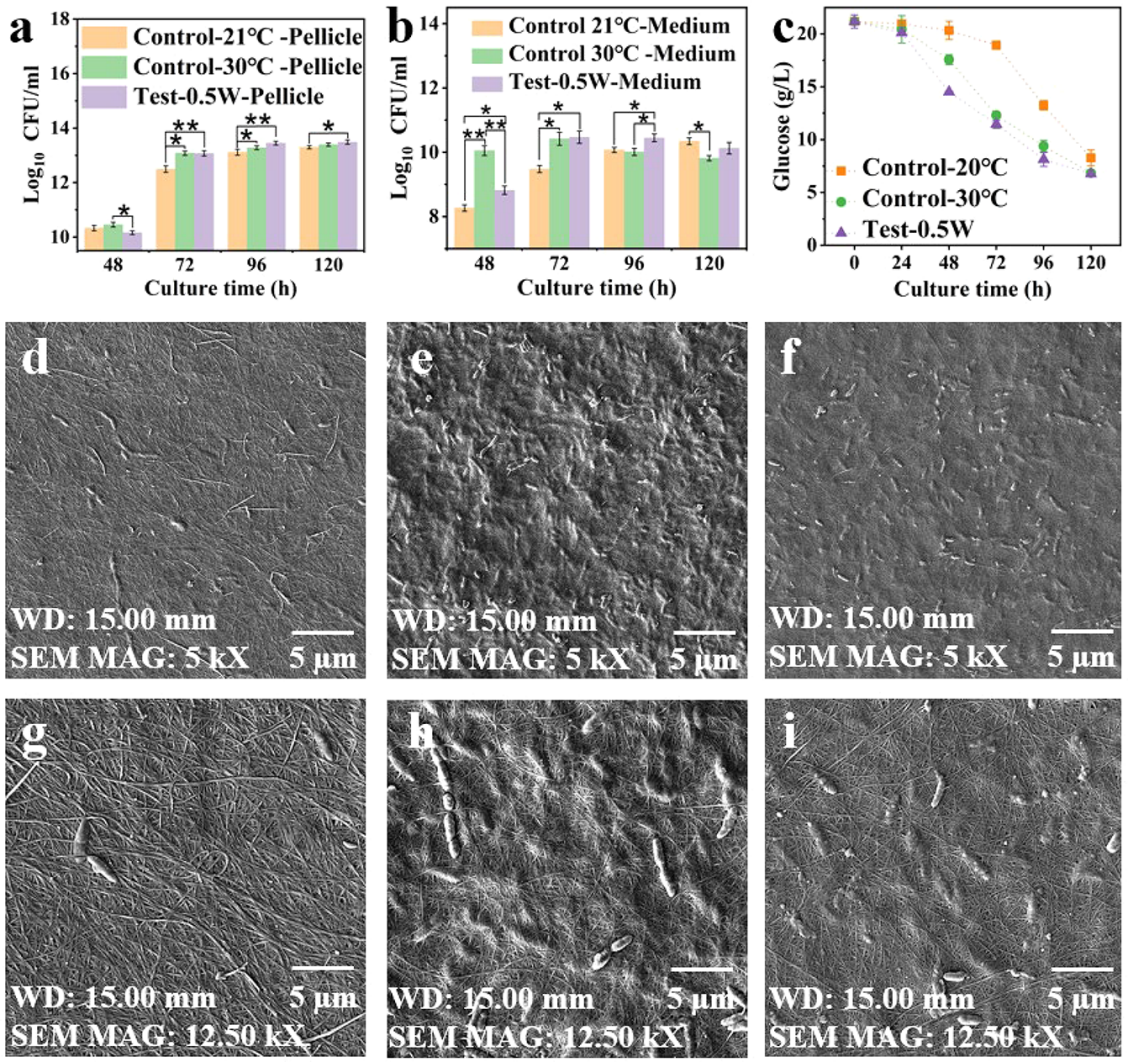
Effects of acoustic wave stimulation on *K. xylinus* growth, glucose consumption, and microstructure of BC pellicles: (a, b) CFU count of bacterial cellulose after 120 h of culture within (a) BC pellicle and (b) culture medium. (c) Glucose concentration in the culture medium measured over time, showing the consumption profile during culture. (d–f) SEM images of BC pellicle top surfaces at 5k magnification: (d) Control group at 21 °C, (e) Control group at 30 °C, (f) SAW-treated group cultured using 0.50 W SAW driver. (g–i) SEM images of BC pellicle top surfaces at 12.5k magnification: (g) Control group at 21 °C, (h) Control group at 30 °C, (i) SAW-treated group cultured using 0.50 W SAW driver. Statistical significance is indicated as follows: *p* ≤ 0.05 (*), *p* ≤ 0.01 (**), *p* ≤ 0.001 (***), and ns = not significant (*p* > 0.05).

In static cultures, BC pellicles are formed at the air–liquid interface, with the lower layer contacting the medium and the upper layer exposed to air.^17,41,42^ As the pellicle thickens, it increasingly obstructs oxygen diffusion into the medium, limiting the availability for the aerobic *K. xylinus*. Consequently, active cellulose production primarily occurs at the oxygen-rich top layer of the pellicle. As oxygen diffusion diminishes with increasing pellicle thickness, bacterial activity becomes progressively confined to this superficial region.^43^ This phenomenon explains why, in the control group, the number of viable cells in the culture medium peaked at 72 h and then gradually declined as more bacteria stayed in the BC pellicle.

Conversely, in the SAW-treated culture medium, bacterial counts peaked between 72 and 96 h, declining only after 120 h. As illustrated in Figure 1, SAW-induced acoustofluidics enhanced the microenvironment by improving nutrient distribution and increasing the dissolved oxygen concentration, thereby facilitating bacterial proliferation and delaying cell viability loss. The sustained bacterial counts in the SAW-treated medium, relative to those of the 30 °C control group, suggest that acoustic waves maintain favorable growth conditions even at later cultivation stages. This benefit is particularly pronounced compared to the 21 °C static control group, where the bacterial population in the medium increased steadily but slowly throughout the 120 h culture, reflecting its suboptimal growth at the ambient temperature. These results demonstrate that acoustic waves can compensate for lower temperatures by creating a more conducive microenvironment for aerobic bacterial activity.

The CFU data from BC pellicles (Figure 2b) showed that the 30 °C static control group maintained the highest bacterial count within 48 h, attributable to efficient oxygen availability at the air–medium interface, supporting rapid metabolism.^44^ However, as the pellicle thickening progressively limited oxygen diffusion, the total bacterial count within this control pellicle fell below that of the SAW-treated group after 72 h. The SAW-treated group, though operated at room temperature, showed a distinct trend that the bacterial counts in the pellicle increased steadily after 48 h, surpassing the control group between 96 and 120 h. This suggests that SAW stimulation enhanced oxygen and nutrient transfer, mitigating the diffusion limitations imposed by the thickening pellicle. SAW-generated acoustic streaming improved the oxygen distribution at the pellicle–medium interface and within the pellicle, sustaining bacterial activity for longer periods. Glucose consumption rates (Figure 2c) corroborate these observations. The more rapid glucose depletion in the SAW-treated medium, despite being at an ambient temperature of ∼21 °C (suboptimal for *K. xylinus*), indicates higher metabolic activity and growth compared to those of the control groups. This further supports that SAW-induced acoustic streaming enhances the *K. xylinus* growth even at ambient temperature.

Morphologies obtained from SEM images confirmed the role of SAWs in enhancing the bacterial growth. At a magnification of 5000×, the 21 °C static control (Figure 2d) showed sparsely distributed, loosely packed bacterial cells (bright, elongated structures), while the 30 °C static control (Figure 2e) exhibited more abundant bacteria, reflecting optimal temperature conditions. In contrast, the SAW-treated sample (Figure 2f), cultured at 21 °C, showed a high density of bacterial cells, indicating that the SAW activation mitigates the lower temperature limitations. At 12,500× magnification, differences in fiber morphology and bacterial distribution were more pronounced. The 21 °C control (Figure 2g) showed a visually loose and discontinuous BC network with sparsely distributed bacterial cells. The 30 °C control (Figure 2h) appeared to form a more continuous and uniform pellicle, while the SAW-treated sample (Figure 2i) showed a similarly uniform and interconnected network with evenly distributed bacterial cells. These qualitative observations highlight the dual effect of SAWs in promoting bacterial growth and increasing BC pellicle density, even at non-optimal temperatures.

### 3.2 BC Yield Modulation with SAWs

The wet weights of BC pellicles increased steadily under all the conditions with cultivation time (Figure 3a). From 48 to 120 h, particularly at later stages (96–120 h), the SAW-treated group exhibited significantly higher wet weights than the 21 °C static control. Although the 30 °C static control group initially showed the highest wet weight, the SAW-treated group surpassed it after 120 h. These results suggest that SAW-induced agitation enhanced cellulose production by promoting efficient bacterial activity and fiber network formation, even at the suboptimal temperature of 21 °C, underscoring the capacity of SAWs to mitigate low-temperature limitations on the *K. xylinus*.

**Figure 3:**
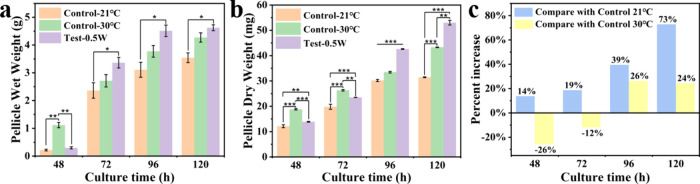
Wet weights (a) and dry weights (b) of BC pellicle. (c) Growth rates of wet weights of pellicles in the SAW-treated group compared with that in the control group. Statistical significance is indicated as follows: *p* ≤ 0.05 (*), *p* ≤ 0.01 (**), *p* ≤ 0.001 (***), and ns = not significant (*p* > 0.05).

The dry weight of BC pellicles followed a similar trend, further corroborating these observations (Figure 3b). After 120 h of cultivation, the SAW-treated group reached a dry weight of 53.33 ± 0.12 mg, compared with 31.47 ± 0.17 mg for the 21 °C static control group and 43.30 ± 0.12 mg for the 30 °C static control group. Although the increase in yield was modest (about 22% higher than 30 °C control and ∼70% higher than 21 °C control), the SAW-treated samples consistently exhibited greater cellulose accumulation, indicating that acoustic stimulation can reproducibly enhance BC biosynthesis at ambient temperature. These findings highlight the dual benefits of SAW application, i.e., promoting bacterial growth and enhancing cellulose yield. The increased dry weight indicates a higher cellulose content, suggesting that acoustic waves facilitate polymerization processes which are essential for cellulose production. This effect is likely influenced by SAW-induced acoustic streaming, which could enhance the aeration around bacterial cell surfaces.

BC production was substantially enhanced under SAW treatment compared to both 21 and 30 °C static control groups (Figure 3c). Compared to the 21 °C static control, SAW agitation achieved a 73 wt % increase in BC production after 120 h. Even compared to the 30 °C static control group, SAW treatment demonstrated a 24 wt % increase by the same time point, highlighting its efficacy at ambient temperatures. Notably, although early stage production (60–72 h) under SAW treatment was marginally lower than the 30 °C control group, SAW stimulation provided a more sustained enhancement of bacterial activity and cellulose production over extended fermentation periods. This characteristic is particularly advantageous for industrial applications requiring a prolonged cultivation. Morphological analysis (see the Supporting Information Figure S4) revealed that pellicles from the SAW-treated group were approximately 1.5 times thicker and more uniform than those from the 30 °C static control group.

### 3.3 Modulation of Cellulose Properties Using SAWs

Analysis of fiber widths and surface roughness in BC pellicles using an AFM revealed distinct structural differences among culture conditions. The mean fiber width measurements indicated that the SAW-treated group exhibited significantly narrower fibers than the 21 °C static control, whereas no statistically significant difference was observed compared to the 30 °C static control group (Figure 4a). This suggests that SAW activation, even operated at the ambient temperature (∼21 °C), helps maintain a microstructural quality comparable to that achieved at the optimal 30 °C. While fiber width alone does not confirm alignment, it is hypothesized that SAW-induced microvibrations influence cellulose bundling, potentially promoting uniform fiber deposition at the ambient temperature.

**Figure 4:**
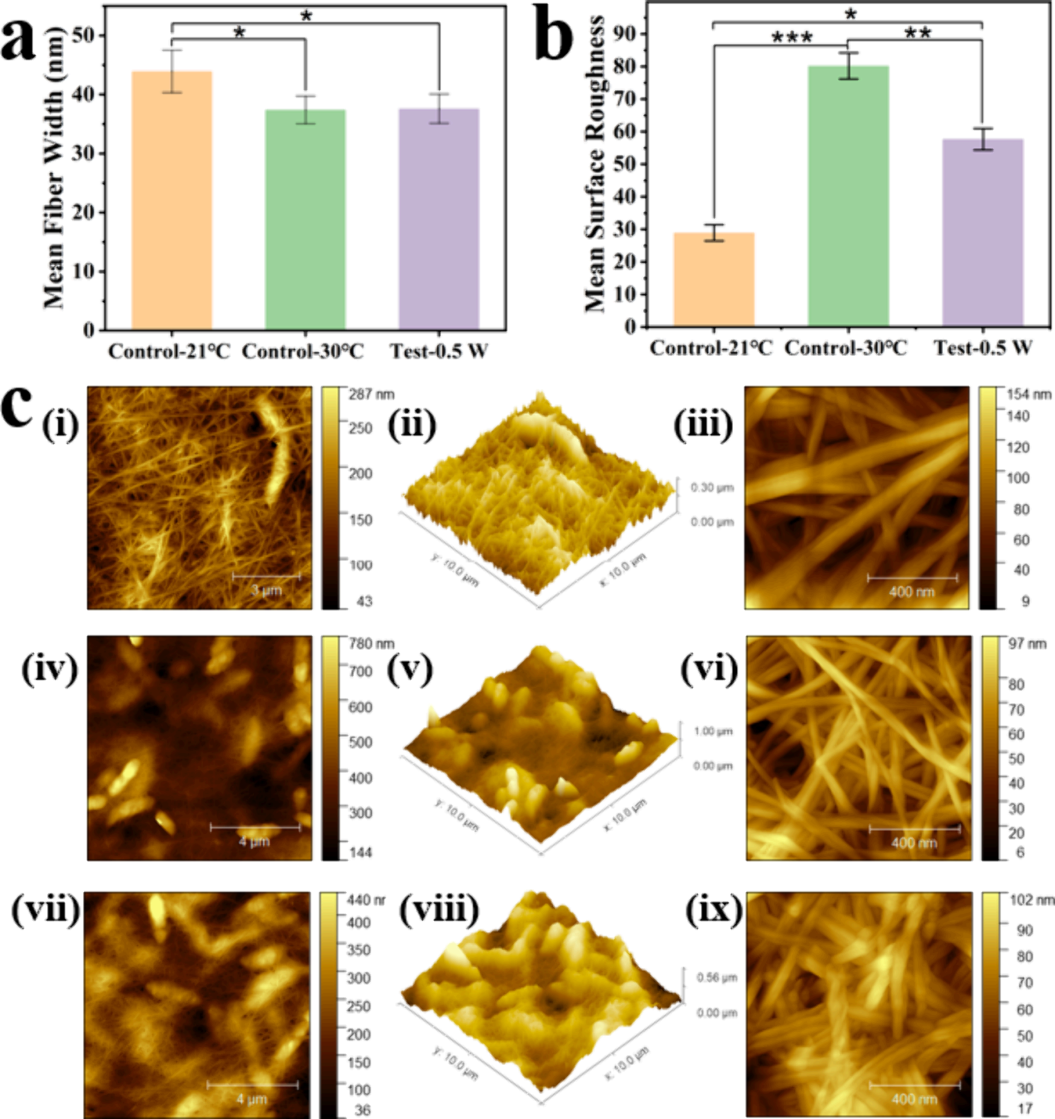
Surface morphology and nanostructural characterization of BC pellicles under different culture conditions: Average fiber width (a) and roughness (b) of BC pellicle under different culture conditions; (c) AFM Surface characterization 2D and 3D images at 10 μm × 10 μm of BC pellicle: (i, ii) Control 21 °C, (iv, v) Control 30 °C, (vii, viii) Test 0.50 W SAW-treated; AFM surface characterization 2D image at 1 μm × 1 μm of BC pellicle: (iii) Control 21 °C, (vi) Control 30 °C, (ix) Test 0.50 W SAW-treated. Statistical significance is indicated as follows: *p* ≤ 0.05 (*), *p* ≤ 0.01 (**), *p* ≤ 0.001 (***), and ns = not significant (*p* > 0.05).

Surface roughness analysis from AFM results (Figure 4b) showed clear distinctions, where the SAW-treated and 30 °C static control groups exhibited higher roughness values than the 21 °C static control group, indicating enhanced structural complexity and surface features due to acoustic stimulation and optimal temperature. Increased surface roughness is often correlated with better surface hydrophobicity for the BC pellicles,^45^ an effect previously observed with ultrasonic agitation.^46^ Thus, SAW treatment not only boosts BC yield but also increases the surface roughness of BC pellicles. Such improved surface morphology is desirable for biomedical applications, composite fabrication, and functional surface modifications, potentially enhancing cell adhesion, interfacial bonding, and surface activity.^47,48^

AFM analysis (Figure 4c) provided detailed insights into the morphological properties of BC pellicles. Over a 10 μm × 10 μm scan area, the surface of the 21 °C static control group appeared sparse and irregular with less densely packed fibers. In contrast, the 30 °C static control group showed more aligned and uniformly distributed fibers, reflecting optimal temperature effects. Similarly, the SAW-treated group displayed compact and well-structured fiber networks with highly aligned fibers and pronounced structural features, comparable to those observed in the 30 °C static control group. At a 1 μm × 1 μm resolution, microstructural details were more evident, with the SAW-treated group demonstrating superior surface uniformity compared to the 21 °C static control group. These results suggest that SAW treatment promoted a more compact and organized fiber network structure compared with the 21 °C static control, which is associated with acoustic streaming-induced microflows within the culture medium.

XRD analysis (Figure 5a) confirms that all of the BC samples exhibited characteristic peaks corresponding to the (100), (010), and (200) crystallographic planes of cellulose. Crystallinity indices were similar across groups, i.e., 74.8% (21 °C control), 76.5% (30 °C control), and 75.6% (SAW-treated). These values are within the typical range (70–80%) reported for *K. xylinus*-derived BC under various cultivation and processing conditions.^49,50^ Minor variations among the different groups are likely attributable to natural differences in bacterial strain activity, culture duration, purification, and drying processes rather than SAW treatment. Both the SAW-treated and 30 °C static control samples showed slightly sharper peaks and higher intensities, particularly for the (200) plane, compared to the 21 °C static control group. This indicates that the SAW treatment group at ambient temperature maintains the intrinsic crystallinity of BC without deterioration in its structural order. The small numerical differences are not statistically significant and are presented here to support the consistency of the crystallographic results under different cultivation conditions.

**Figure 5:**
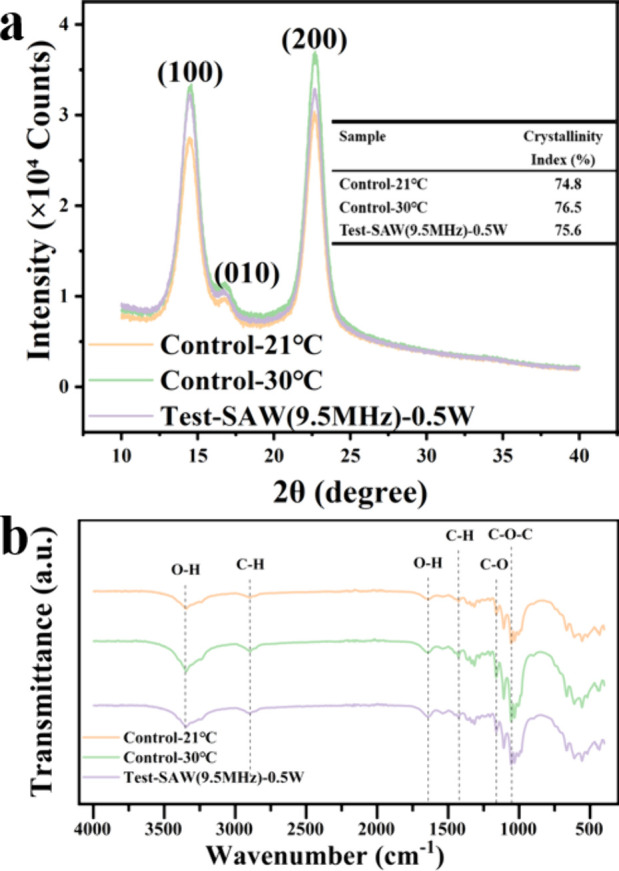
Crystallinity and molecular structure of BC pellicles under different culture conditions: (a) XRD patterns of BC pellicle. (b) FT-IR analyses of BC pellicle.

Fourier transform infrared spectroscopy (FTIR) analysis (Figure 5b) further confirmed the characteristic cellulose functional groups (O–H, C–H, and C=O stretching vibrations) in all of the samples. The transmittance intensities of these peaks were slightly stronger in the SAW-treated and 30 °C static control groups compared with those of the 21 °C static control group. Overall, these findings indicate that the SAW stimulation group preserved the crystalline and molecular characteristics of BC similar to those obtained under conventional 30 °C incubation. The potential energy benefits of operating at ambient temperature will be discussed in the following section.

The mechanical properties of the SAW-treated BC samples, including Young’s modulus, tensile strength/stress, and elongation at break, were further obtained using the tensile tests (Figure S5 in the Supporting Information). All of the results showed that the SAW-assisted BC samples showed comparable results compared to those of the control groups. In brief, all of the above results demonstrate that acoustic wave treatment operated at ambient temperature maintains the structural and morphological integrity of bacterial cellulose without compromising its mechanical performance. Although no significant enhancement in molecular organization was observed, SAW stimulation achieved BC yield and quality similar to those of the conventional 30 °C cultivation process. This indicates that SAW-assisted cultivation provides a reliable and effective approach for bacterial cellulose production under ambient conditions.

### 3.4 Mechanistic Insights into SAW Influence on BC Biosynthesis

BC biosynthesis is a complex and multifactorial process involving bacterial metabolism, quorum sensing, cellulose synthase activity, and environmental regulation. These processes are strongly influenced by mass transport and local mechanical cues within the culture medium.^51^ It is therefore plausible that the observed enhancement of the BC yield under SAW stimulation arises from several interrelated mechanisms.

SAWs can generate acoustic streaming and microstreaming flows which enhance nutrient diffusion and metabolite transport within the culture medium.^52^ This improvement in mass transfer alleviates one of the main limitations of static cultivation, namely, the restricted oxygen and nutrient supply, thereby supporting bacterial proliferation and sustained cellulose synthesis. In this study, the generation of acoustic streaming under SAW excitation was experimentally demonstrated by introducing 5 μm polypropylene particles into the culture medium, which exhibited clear flow trajectories driven by the acoustic field (shown in the Supporting Information, Video S1), thereby confirming the presence of SAW-induced streaming.

Low-intensity ultrasound has been shown to promote microbial proliferation by loosening cell aggregates, enhancing nutrient transfer, and increasing membrane permeability, thereby accelerating metabolic activity and metabolite production.^33^ In contrast, high-intensity ultrasound or prolonged exposure to ultrasound can disrupt cell walls, damage membranes, and inactivate cells, often through cavitation and shear forces.^53^ Recent work using thin-film-based SAW devices demonstrated a similar biphasic response. For examples, SAW powers below ∼2.5 W enhanced the growth of *Escherichia coli* and *Staphylococcus aureus*, whereas higher powers led to inactivation, which was attributed to acoustic streaming, microstreaming-induced shear stresses, and localized heating at the liquid–solid interface.^19^

These findings suggest that SAWs could influence BC production not only by altering nutrient transport and oxygenation in the medium (two critical parameters for the BC yield) but also by modulating bacterial motility and quorum sensing dynamics, which govern cellulose synthase expression. Thus, the observed effects of SAW on BC yield may arise from a combination of enhanced mass transfer and mechanical and thermal stresses, providing a plausible mechanistic basis beyond empirical observations. However, further research needs to be performed to verify these mechanisms.

### 3.5 On-site Energy Consumption and Direct GHG Emissions

A significant advantage of SAW-activated BC production at ambient temperature is the substantial reduction in energy consumption and carbon emissions compared to the conventional incubation at 30 °C for 120 h. The SAW-treated group was cultured at an ambient base temperature of 20 °C, with the medium experiencing an ∼1 °C rise due to the acoustic stimulation. In contrast, the 30 °C static control group required a fixed-temperature incubator (rated power 250 W), which consumes considerable energy to maintain the temperature of its entire larger volume. SAW devices, however, were operated on a smaller, localized, and precisely controlled scale, using acoustic waves to directly influence bacterial activities.

In our experimental setup, the incubator (capacity of 16 beakers) exhibited an operating power of approximately 84 W to maintain 30 °C. Over 120 h, this translated into an estimated total energy consumption of 10 kWh and carbon emissions of 4.0 kg of CO_2_e. Consequently, the per-beaker energy consumption and carbon footprint for incubator-based cultivation were estimated at 0.625 kWh and 0.25 kg of CO_2_e, respectively. Conversely, the SAW-treated group, over the same 120 h cultivation period, consumed an estimated 0.060 kWh, rendering it approximately 10-fold more energy-efficient. Correspondingly, the SAW treatment generated an estimated 0.024 kg CO_2_e, achieving a >90% reduction in carbon emissions compared to the incubator cultivation.

Normalizing for BC yield (dry weight) over 120 h further highlighted these disparities. Energy consumption and carbon emissions per gram of BC for the SAW-treated group were estimated at 4.5 × 10^–4^ kWh and 1.1 × 10^–3^ kg CO_2_e, respectively. For the incubator-based method, these values were 5.8 × 10^–3^ kWh and 1.4 × 10^–2^ kg CO_2_e. Thus, the incubator method consumed nearly 13 times more energy and produced over 12 times more carbon emissions per gram of BC than the SAW treatment.

Unlike incubator-based methods reliant on energy-intensive temperature control, SAW devices (consuming only 0.50 W) can be operated at a localized scale (e.g., lab-on-a-chip) to create a microenvironment conducive to bacterial growth. This enables SAWs to achieve comparable bacterial activity and BC yields at a fraction of the energy cost, presenting a viable and sustainable alternative for small-scale BC production. By overcoming the limitations of low-temperature cultivation, SAW technology offers a highly energy-efficient and environmentally friendly solution for BC yields without compromising productivity. These findings underscore the potential of SAW treatment as a sustainable alternative for BC production at ambient temperature, particularly for conditions where a smart microsystem, energy efficiency, and environmental impact are critical considerations. However, we should address that the current comparison simply reflects laboratory-scale experiments rather than industrial production. Further optimization and adjustment will be required before SAW devices can be scaled up, particularly to address challenges in equipment integration and process implementation.

## 4 Summary

This study pioneers the application of SAW agitation to enhance the production of BC. We demonstrate that SAW integration into the cultivation process significantly augments *K. xylinus* growth at ambient temperature (21 °C), although it should be noted that the present comparison was limited to the first 120 h of cultivation. The substantial reductions in energy consumption (∼10-fold) and carbon emissions (>90%) achieved with SAW treatment underscore its clear advantages compared to the conventional 30 °C static incubation case. Importantly, the SAW-treated group yielded BC with comparable quantities and material properties to those from the 30 °C static control. These findings highlight the SAW stimulation as a sustainable and efficient advancement in static BC production methods, potentially obviating the need for energy-intensive constant-temperature incubation while maintaining high productivity and superior material characteristics. Further studies are required to evaluate whether the advantages of SAW stimulation can be sustained during the later stages of cultivation (120–336 h). Moreover, the scalability of SAW systems could be explored through array, modular, and tunable device configurations for larger-area or continuous BC production. The film quality of SAW-treated BC was similar to that obtained under the conventional 30 °C cultivation, revealing that acoustic stimulation maintained the desired structural uniformity and functionality while improving energy-related sustainability. Combined with its inherent biocompatibility, this makes SAW-treated BC highly suitable for demanding biomedical applications such as wound healing, tissue engineering, and controlled drug delivery systems. Overall, SAW-treated static cultivation represents a highly sustainable and energy-efficient approach to BC production at ambient temperatures, heralding a promising technological advancement for the future of bio-based materials manufacturing.

## Supplementary Material



## Notes

Experimental setup, heating effect analysis, BC pellicle growth images under different conditions, mechanical testing results, and demonstration videos of acoustic streaming generated by the SAW device (DOCX)
