# Supplementary material for: Acoustofluidics Powered Synthesis of Bacterial Cellulose
Source: ACS Sustain Chem Eng. 2025 Nov 18;13(47):20402–11. doi: 10.1021/acssuschemeng.5c07599 (PMC12673587; doi:10.1021/acssuschemeng.5c07599)
Supplement: Supplementary file 1 [file sc5c07599_si_001.docx]

**Supplementary Information**

Acoustofluidics Powered Synthesis of Bacterial Cellulose

Jikai Zhang,^1^ Katie Gilmour,^2^ Meng Zhang,^2, *^ Yunhong Jiang,^2^ Peter Arnold,^1^ Huiling Ong,^1^ Qiang Wu,^1^ Maryam Parsa,^1^ Ran Tao,^3, *^ Jingting Luo,^3^ and Yongqing Fu^1,^ *

1. School of Engineering, Physics and Mathematics, Northumbria University at Newcastle, Newcastle upon Tyne, NE1 8ST, UK
2. Living Construction Group, School of Geography and Natural Sciences, Northumbria University at Newcastle, Newcastle upon Tyne, NE1 8ST, UK
3. Key Laboratory of Optoelectronic Devices and Systems of Ministry of Education and Guangdong Province, College of Physics and Optoelectronic Engineering, Shenzhen University, Shenzhen, 518060, China

* Corresponding Author, Prof. Meng Zhang, e-mail: [meng.zhang@northumbria.ac.uk](mailto:meng.zhang@northumbria.ac.uk); Dr. Ran Tao, E-mail: [ran.tao@szu.edu.cn](mailto:ran.tao@szu.edu.cn); Prof. Richard Yongqing Fu, email: [Richard.fu@northumbria.ac.uk](mailto:Richard.fu@northumbria.ac.uk)

Summary

This Supporting Information file contains 5 figures (S1–S5) and 2 videos (V1a and V1b). Figures include the SAW device setup, heating effect analysis, BC pellicle growth under different conditions, and mechanical testing results. Videos V1a and V1b demonstrate acoustic streaming generated by SAW excitation, shown from side and top views, respectively.


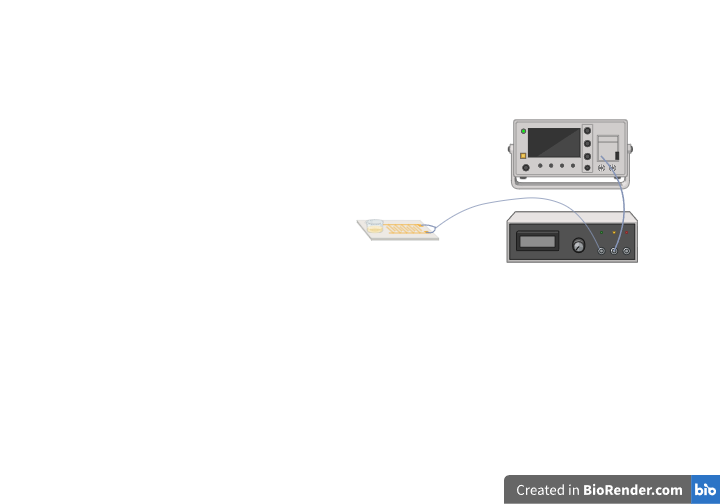


**SAW device**

**Amplifier**

**signal generator**

Fig. S1. SAW device and experiment setup.


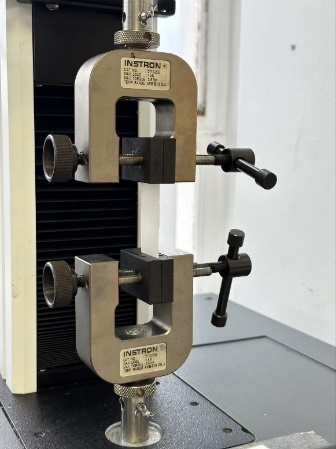


Fig. S2. Photograph of mechanical tests on bacterial cellulose.


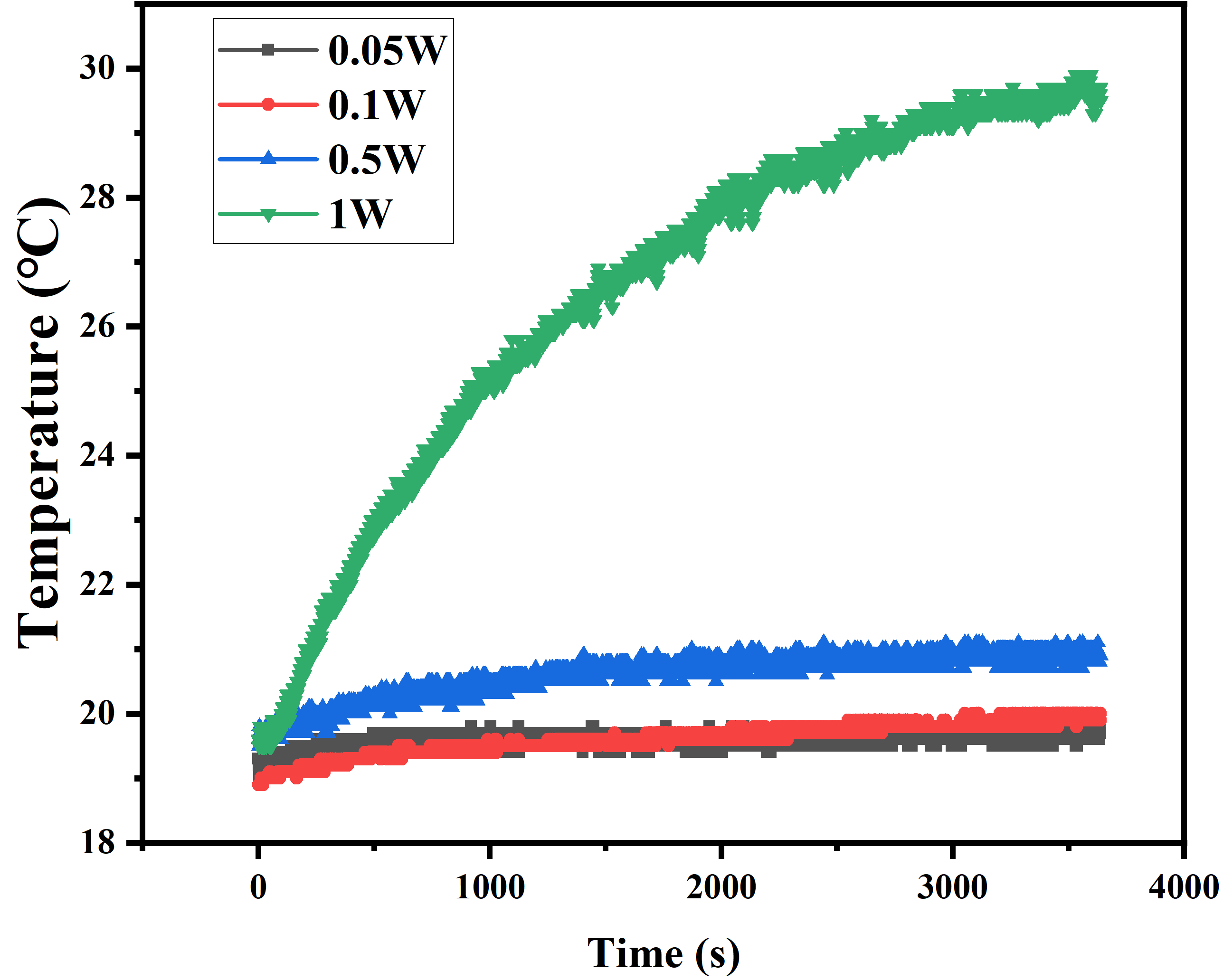


Fig. S3. The heating effects induced by SAWs in the culture medium at powers of 0.05 W, 0.1 W, 0.5 W and 1 W within an incubator with a temperature of 20 °C.


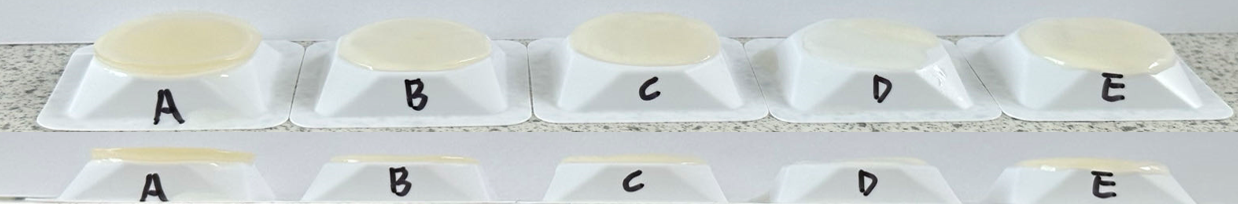


Fig. S4. Images of the harvested BC pellicle. The SAW-treated hydrated BC pellicle after (A) 120 hrs, (B) 96 hrs, (C) 72 hrs, (D) 60 hrs; Control group at 30°C hydrated BC pellicle after (E) 120 hrs


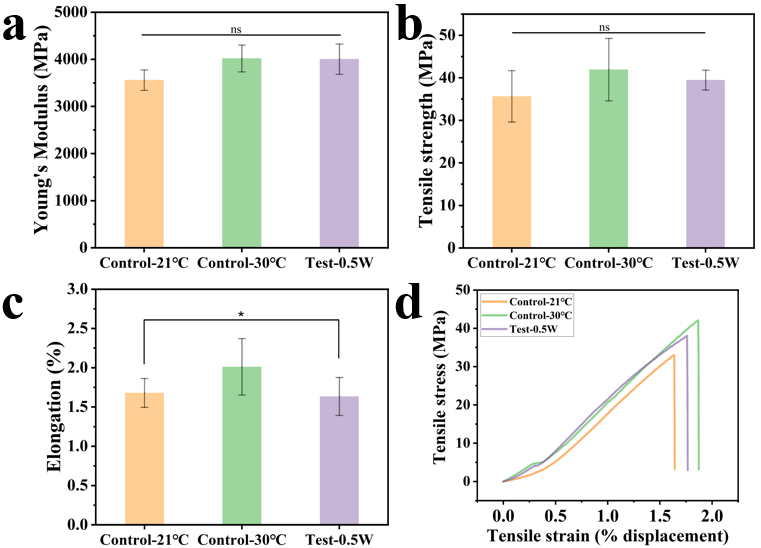


Fig. S5. Mechanical properties of BC pellicles under different culture conditions: (a) Young's modulus results; (b) Tensile strength results; (c) Elongation results; (d) The representative stress strain curves from tensile testing.

The mechanical properties for the BC samples are shown in Figs. S5a-S5d. Results indicate that the SAW treatment did not significantly affect their overall mechanical performance. The Young’s moduli and tensile strengths show no significant differences across all the groups. Notably, in terms of elongation, although the SAW-treated group shows clear differences with the 21°C control, but the statistical difference with those of the 30°C control group is insignificant. The stress–strain curves also reflect similar deformation behaviours among all the groups as shown in Fig. S5d. These results reveal that while SAW treatment significantly enhanced BC proliferation, it did not result in significantly enhanced mechanical properties.

Video S1. Acoustic streaming generated by SAW excitation, visualised using 5 μm polypropylene particles suspended in the culture medium: (a) side view video; and (b) top view video.


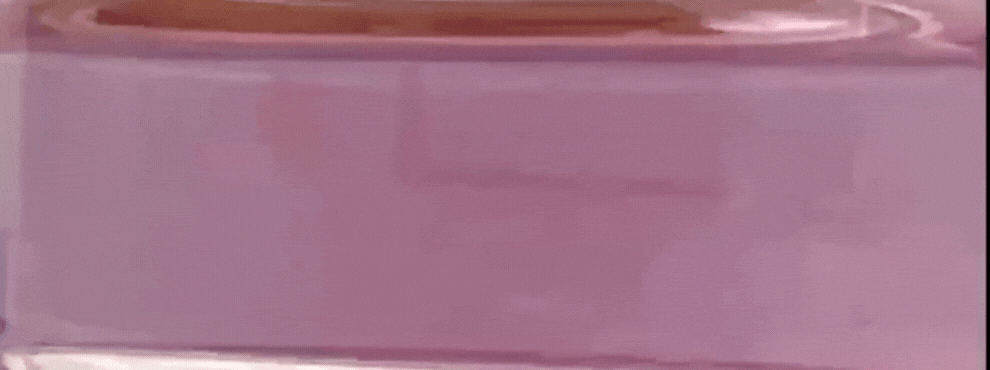
(a)


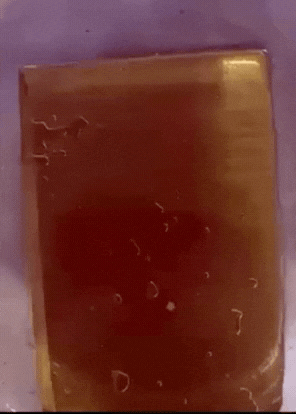
(b)
